# Long-Read–Based Hybrid Genome Assembly and Annotation of Snow Algal Strain CCCryo 101-99 (cf. *Sphaerocystis* sp., Chlamydomonadales)

**DOI:** 10.1093/gbe/evae140

**Published:** 2024-06-28

**Authors:** Ozan Çiftçi, Athanasios Zervas, Stefanie Lutz, Helen Feord, Christoph Keusching, Thomas Leya, Martyn Tranter, Alexandre M Anesio, Liane G Benning

**Affiliations:** Interface Geochemistry, GFZ German Research Centre for Geosciences, Potsdam, Germany; Department of Environmental Science, Aarhus University, Roskilde, Denmark; Interface Geochemistry, GFZ German Research Centre for Geosciences, Potsdam, Germany; Plant-Soil Interactions, Department of Agroecology and Environment, Agroscope, Zurich, Switzerland; Interface Geochemistry, GFZ German Research Centre for Geosciences, Potsdam, Germany; Interface Geochemistry, GFZ German Research Centre for Geosciences, Potsdam, Germany; Fraunhofer Institute for Cell Therapy and Immunology, Branch Bioanalytics and Bioprocesses IZI-BB, Potsdam, Germany; Department of Environmental Science, Aarhus University, Roskilde, Denmark; Department of Environmental Science, Aarhus University, Roskilde, Denmark; Interface Geochemistry, GFZ German Research Centre for Geosciences, Potsdam, Germany; Department of Earth Sciences, Freie Universität Berlin, Berlin, Germany

**Keywords:** genomes, snow, cryophilic, algae, climate change, chlorophyta

## Abstract

Polar regions harbor a diversity of cold-adapted (cryophilic) algae, which can be categorized into psychrophilic (obligate cryophilic) and cryotrophic (nonobligate cryophilic) snow algae. Both can accumulate significant biomasses on glacier and snow habitats and play major roles in global climate dynamics. Despite their significance, genomic studies on these organisms remain scarce, hindering our understanding of their evolutionary history and adaptive mechanisms in the face of climate change. Here, we present the draft genome assembly and annotation of the psychrophilic snow algal strain CCCryo 101-99 (cf. *Sphaerocystis* sp.). The draft haploid genome assembly is 122.5 Mb in length and is represented by 664 contigs with an N50 of 0.86 Mb, a Benchmarking Universal Single-Copy Orthologs (BUSCO) completeness of 92.9% (*n* = 1,519), a maximum contig length of 5.3 Mb, and a guanine-cystosine (GC) content of 53.1%. In total, 28.98% of the genome (35.5 Mb) contains repetitive elements. We identified 417 noncoding RNAs and annotated the chloroplast genome. The predicted proteome comprises 14,805 genes with a BUSCO completeness of 97.8%. Our preliminary analyses reveal a genome with a higher repeat content compared with mesophilic chlorophyte relatives, alongside enrichment in gene families associated with photosynthesis and flagella functions. Our current data will facilitate future comparative studies, improving our understanding of the likely response of polar algae to a warming climate as well as their evolutionary trajectories in permanently cold environments.

**Significance**

In polar regions, cold-adapted algae thrive and bloom, yet genomic studies on these organisms are scarce. This is hindering our understanding of their adaptive mechanisms in the face of climate change. Here, we provide the first genome assembly of the psychrophilic snow algal strain CCCryo 101-99, revealing a higher repeat content compared with mesophilic relatives, and enrichments in gene families crucial for photosynthesis and flagellar functions. This genome data will allow further comparative analyses and help us obtain critical insights into the adaptive strategies of polar algae and their responses to a changing climate.

## Introduction

Polar and alpine regions are characterized by extreme environmental conditions with snow and ice covering ∼12% of the Earth's land surface. Significant yet not well-studied components of the polar biota are cold-adapted eukaryotic snow and glacier ice algae, which are the main primary producers in these ecosystems. They shape local biogeochemical cycles and affect albedo through their pigmentation, thus enhancing melting (Lutz et al. 2016; Chevrollier et al. 2023). On semipermanent and permanent snow fields, different snow algae phenotypes bloom during the summer melt seasons, despite the harsh conditions characterized by low temperatures, high irradiation, and oligotrophic nutrient levels. Snow algae produce green, yellow, orange, and red algal blooms on snow fields worldwide (Lutz et al. 2016; Segawa et al. 2018). The ever-lengthening melt seasons, in particular, in future warming climate scenarios, will lead to an expansion of the habitat extent of snow algae, further exacerbating glacier melt (Lutz et al. 2014; Segawa et al. 2018).

Various chlorophyte algae species belonging to the genera *Chlainomonas*, *Chlamydomonas*, *Chlorominima*, *Chloromonas*, *Limnomonas*, *Microglena*, *Raphidonema*, *Sanguina*, and *Scotiella* have been described from snow habitats, and their ecology, physiology, biogeographical distribution, and taxonomic diversity have been characterized (e.g. Remias et al. 2018; Procházková et al. 2019; Tesson and Pröschold 2022; Novis et al. 2023 and references therein). Molecular studies, particularly metabarcoding and metagenomic approaches, have only recently provided the first critical insights into polar biodiversity and evolution and adaptation to permanently cold environments (Clark et al. 2023). However, most work has focused on marine settings, and there are only a few available psychrophilic algae genomes (Blanc et al. 2012; Mock et al. 2017; Zhang et al. 2020, 2021; Hulatt et al. 2024). This hampers our ability to understand their evolutionary history, metabolic potential, and cellular responses and to better address their potential roles in future global warming climate scenarios. Here, we present extensive DNA and RNA sequencing (RNA-seq) data, a long-read–based draft assembly, annotations, and preliminary functional enrichment results of the genome of the psychrophilic snow algal strain CCCryo 101-99 (cf. *Sphaerocystis* sp.).

## Results and Discussion

### Genome Assembly

DNA sequencing using Illumina, PacBio, and Nanopore generated 31,984,466 (7.55 Gb), 1,986,964 (14.1 Gb, read N50: 12.8 kb), and 1,591,524 (1.9 Gb, read N50: 2 kb, max: 400 kb) reads, respectively. For the estimated genome size of 160 Mb, PacBio and Nanopore reads cumulatively provided 98× sequencing depth (86× and 12×, respectively). For error correction with Illumina short reads, 21,366,832 (3.7 Gb) trimmed and quality-filtered reads were mapped onto the long-read assembly with a mapping rate of 98.8%. We compared the metrics and qualities of the assemblies produced by different error correction strategies and assemblers (supplementary table S1, Supplementary Material online). Canu hybrid long-read assembly corrected with Illumina short reads was the longest and most contiguous and had the highest mapping rate and BUSCO coverage and smallest number of assembly errors. An initial error correction step with long reads decreased the BUSCO coverage and contig N50 metrics and introduced additional assembly errors, so the correction was performed using short reads only.

In contaminant screening, 107 contigs comprising 73% of the total genome length (∼90 Mb) were assigned to Chlorophyta and 8 contigs (1.8 Mb) to Streptophyta, and 557 contigs (∼30 Mb) had no hits. In total, 66 contigs of 380 kb length that were assigned to Actinomycetota and Pseudomonadota were removed. The corrected and filtered haploid draft genome is 122.5 Mb in length represented by 664 contigs (68.3× average read coverage) with an N50 of 0.86 Mb, a BUSCO completeness of 92.9% (complete: 92.9% [single-copy: 92.4%, duplicated: 0.5%], fragmented: 2.5%, missing: 4.6%, chlorophyta_odb10, *n* = 1,519, mode = genome), a maximum contig length of 5.3 Mb, and a guanine-cystosine (GC) content of 53.1% (Table 1). The total genome size was estimated to be around 160 Mb based on *k*-mer counts of Illumina sequencing reads. One contig, 458 kb in length and assigned to Chlorophyta, was identified as a candidate chloroplast genome based on GC content (36.5%) and read coverage (2,586×).

**Table 1:** Genome assembly and annotation statistics of CCCryo 101-99 in comparison with other chlorophyte algae genomes

		Assembly size (kb)	Number of contigs/scaffolds	Longest contig (kb)	BUSCO (%)	N50	L50 (kb)	GC (%)	Repeat content (%)
Assembly	CCCryo 101-99	122,425	664	5,321	92.9	39	855	53.1	29.98
	*Chlamydomonas* sp. UWO241^a^	211,600	2,458	nd	85.0	165	370	60.6	49
	*Limnomonas spitsbergensis* ^b^	260,248	124	10,736	nd	21	3,933	54.1	33.16
	*C. reinhardtii* (v5.5)^c^	111,098	17^d^	9,731	96.5	7	7,780	64.1	21.64

		Number of protein-coding genes	Mean gene length (kb)	Mean protein length (aa)	BUSCO (%)	Mean number of exons per gene	Mean exon length (bases)	Mean number of introns per gene	Mean intron length (bases)
Annotation	CCCryo 101-99	14,805	5.98	632	97.8	7.8	314	7.3	516
	*Chlamydomonas* sp. UWO241^a^	16,325	8.06	nd	nd	nd	1,563	10.1	934
	*Limnomonas spitsbergensis* ^b^	18,277	nd	nd	95.2	6.7	nd	nd	788
	*C. reinhardtii* (v5.6)^c^	16,656	5.48	nd	96.1	nd	nd	7.8	229

nd, not determined.

^a^ Zhang et al. 2021. ^b^Hulatt et al. 2024. ^c^Craig et al. 2021. ^d^17 chromosomes + 37 unassembled scaffolds.

### Genome Annotation

The repeat content of the genome is 28.98% (35.5 Mb), and the most abundant category was unidentified repeats (13.24%; supplementary table S2, Supplementary Material online). Long terminal repeats (LTRs) comprised 8.85% of the genome, and the most abundant LTR family was Gypsy/DIRS1 (8.41%) which has been found in diverse eukaryotic species including green algae (Piednoël et al. 2011). The second most abundant repeat category was long interspersed retrotransposable elements (LINEs, 4.38%), and the most abundant LINE family was L1/CIN4 (2.67%). CCCryo 101-99 genome has a slightly higher repeat content compared with its mesophilic chlorophyte relatives, 22% in *Chlamydomonas reinhardtii* (Payne et al. 2023) and 24.76% in *Volvox carteri* (Prochnik et al. 2010), but not as high as other cold-adapted chlorophytes, 64% and 49% in *Chlamydomonas* sp. ICE-L and UWO, respectively (Zhang et al. 2020, 2021). Increased content of tandem repeats and transposable elements in the genome can potentially be helpful for organisms that survive in extreme habitats by creating genetic diversity that can drive the emergence of new adaptive traits (Schrader and Schmitz 2019).

Altogether, 197 transfer RNA (tRNA) genes were predicted, including one pseudogene and one selenocysteine tRNA gene, as well as 100 ribosomal RNA (rRNA) genes including 6 tandem repeats of the ribosomal DNA (rDNA) cistron on the same contig (18S, ITS1, 5.8S, ITS2, and 28S). These were 5.8 kb each and were separated by approximately 3 kb long intergenic spacers. Five of these six rDNA copies have identical sequences for their whole length, and one additional copy was identified in another contig. Other noncoding RNA (ncRNA) classifications include 84 histone 3′ untranslated region (UTR) stem-loop, 17 spliceosomal RNA (U1, U2, U4, U5, and U6), 15 small nucleolar RNA (R38, R160, SNORD14, SNORD24, U3, U36a, Z157, and Z159), 2 mRNA (mir-191), 1 RNase MRP, and 1 antisense RNA (isrR).

**Fig. 1.**
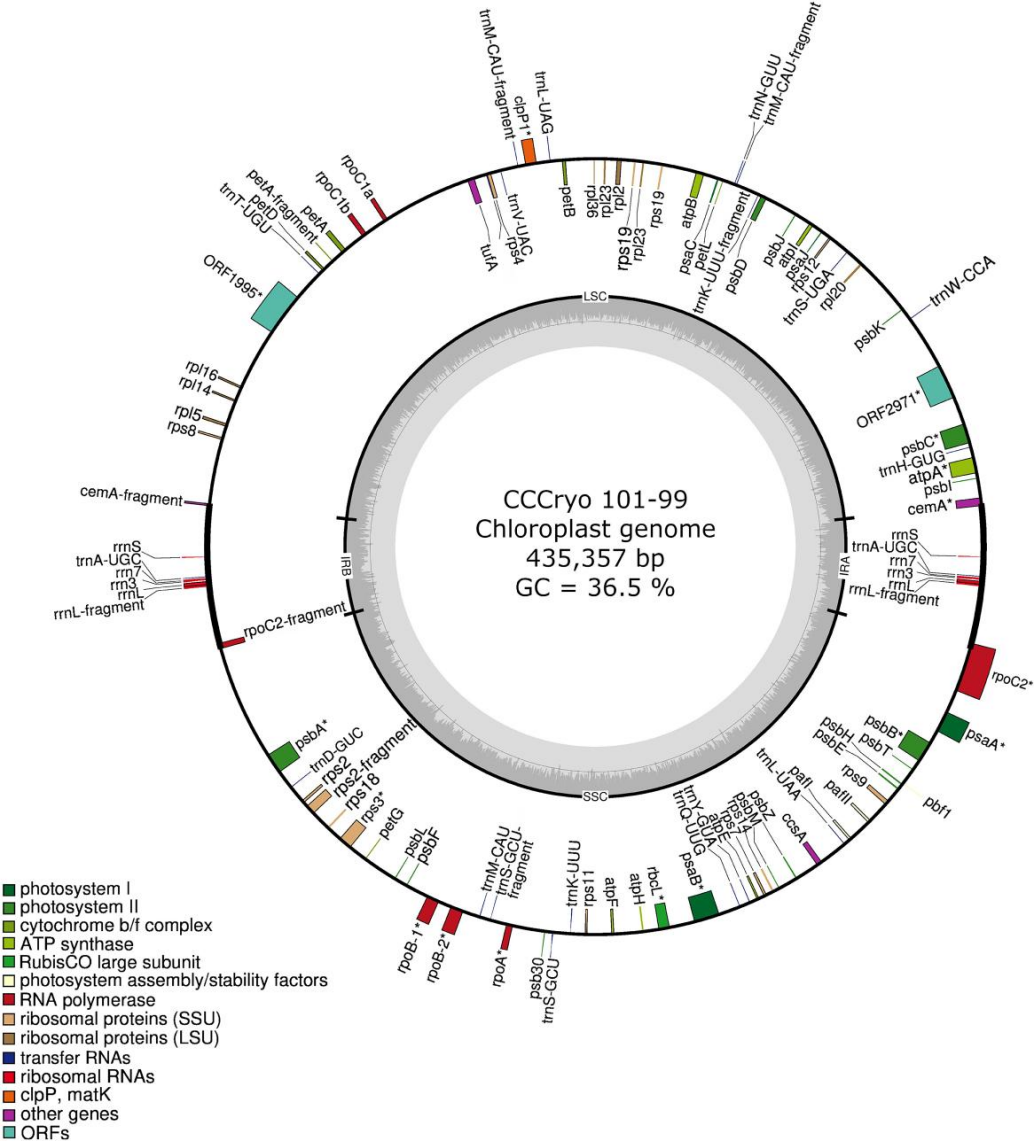
The chloroplast genome of CCCryo 101-99. LSC, long single-copy region; SSC, short single-copy region; IRA, inverted repeat A; IRB, inverted repeat B.

The assembled chloroplast genome is circular, has one large (254 kb) and one small (202 kb) single-copy region, and has an inverted repeat comprising five rRNA and one tRNA genes (Fig. 1). In total, 112 genes were annotated in the chloroplast genome including 10 rRNA and 24 tRNA genes. In addition, four group I and three group II catalytic introns were identified. We performed additional analyses to assemble the mitochondrial genome using 65 Chlamydomonadales mitogenomes as a reference; however, no genes could be annotated on assembled contigs. The mitochondrial genomes of chlorophytes harbor significant diversity in terms of gene content and genome structure (Popescu and Lee 2007). *Chlamydomonas*-like mitochondrial genomes have reduced organizational pattern characterized by small genome size, limited gene content, and the presence of fragmented and scrambled rRNA coding regions (Nedelcu et al. 2000). Our results suggest that CCCryo 101-99 follows this *Chlamydomonas*-like mitochondrial genome evolution.

The Illumina RNA-seq generated 8,748,326 (1.32 Gb) reads. After quality filtering, 4,772,938 (0.72 Gb) reads were mapped onto the genome assembly with a 92.4% mapping rate. Using the RNA-seq mapping as extrinsic evidence, 17,206 transcripts and 14,805 genes were predicted, where 5′ and 3′ UTR regions were identified for 94% of the transcripts, and 99.5% of the genes had both start and stop codons. The BUSCO completeness of the predicted proteome is 97.8% (complete: 97.8% [single-copy: 96.4%, duplicated: 1.4%], fragmented: 0.5%, missing: 1.7%, chlorophyta_odb10, *n* = 1,519, mode = proteome; Table 1).

Genome annotation assigned a protein or domain to 13,707 genes in total (92.5%) (supplementary table S3, Supplementary Material online). The most enriched category (enrichment score = 37.81) in the functional enrichment analysis against the *C. reinhardtii* genome included the Gene Ontology (GO) term “chloroplast thylakoid membrane” and photosynthetic genes (i.e. *psbW*, *petH*, and *rubA*). Duplicates and increased protein accumulation of the photosynthetic genes have been reported (Zhang et al. 2020) in the cold-adapted Antarctic green alga *Chlamydomonas* sp. UWO241 (i.e. compared with the mesophilic relative *C. reinhardtii*). These authors hypothesized that this is an indication of an adaptation to the cold. The second most enriched category in our annotation (enrichment score = 37.29) contained flagellum-associated genes (CFAP58/91/157/221). During its asexual life cycle, CCCryo 101-99 produces motile spores (zoospores) representing young stages of haploid spores. These are not frequently observed in laboratory-grown cultures as they shed their flagella shortly after attaching to surfaces and develop into nonmotile adult spores that divide several times by multiple mitoses and develop into sporangia. Later, young motile zoospores are released from these sporangia to fulfill the asexual life cycle. Nothing is known about their sexual reproduction.

In summary, our analyses on the genome of the snow algal strain CCCryo 101-99 (cf. *Sphaerocystis* sp.) revealed a slightly higher (29.98%) repeat content, compared with the mesophilic chlorophyte relative *C. reinhardtii*. Furthermore, the gene families associated with photosynthesis and flagellar functions were enriched. The former may contribute to the adaptation to cold climates by maintaining a high level of CO_2_ fixation and, thus, optimizing energy production, while the latter may be important for an effective dispersal strategy in snow habitats by maintaining motility in young cell stages. Our draft-assembled snow algal genome and ongoing work on comparative snow algal genomics will allow in-depth comparisons with the increasing number of available genomes. This will improve our understanding of the evolution and diversification of photosynthetic eukaryotes adapted to living in permanently cold climates and their responses to future global warming climate scenarios.

## Materials and Methods

### Strain Description

The snow algal strain CCCryo 101-99 was isolated in 1998 from moss patches along melt streams flowing down from snow fields above on Brøggerhalvoya northwest of Ny-Ålesund, Svalbard, Norway. The strain has been deposited and since then been maintained at the CCCryo Culture Collection of Cryophilic Algae at the Fraunhofer IZI-BB, Potsdam, Germany (Leya 2022). The taxonomic identity of CCCryo 101-99 still is under debate; therefore, for the time being, it is called cf. *Sphaerocystis* sp. According to its phylogenetic marker (*rbcL*), it shows relatedness (data not shown) to the genus *Achoma* (Novis and Visnovsky 2012). Detailed studies are currently in progress. It can be classified as a psychrophilic snow alga as its optimum temperature for growth lies at around 14 °C; its maximum temperature lies above 18 °C (Leya et al. 2009), but above that it starts ceasing growth and eventually dies. A progeny of CCCryo 101-99 had spent 531 d outside the International Space Station in its desiccated cyst stage as part of the BIOMEX experiments and survived (de Vera et al. 2019). CCCryo 101-99 extracts are also used in cosmetic products to guard against aging of the skin caused by extrinsic factors, such as ultraviolet radiation or air pollution, or intrinsic factors such as aging-specific gene expression levels (Stutz et al. 2010). The strain is known to synthesize the antioxidants and pigments: astaxanthin, adonixanthin, canthaxanthin, echinenone, hydroxyechinenone, and saccharose (Leya 2022).

### Cultivation and DNA/RNA Extractions

The cultures for nucleic acid extractions were grown in a 3N Bold's Basal Medium at pH 5.5 and 2 °C (green biomass) or 12 °C (orange biomass induced from green biomass) under axenic conditions and continuous illumination. Genomic DNA (gDNA) was extracted from green biomass using the PowerSoil DNA Isolation kit (QIAGEN, Germany) for Illumina sequencing and the QIAGEN Genomic-tips extraction kit for PacBio sequencing, following the manufacturer's instructions. gDNA extraction for Nanopore sequencing was performed using a cetyltrimethylammonium bromide (CTAB)/phenol extraction of green biomass following Chaux-Jukic et al. (2024). We assessed the size, quantity, and integrity of the extracts on a TapeStation 4150 (Agilent Technologies) and a Qubit 4 Fluorometer (Thermo Scientific) and via gel electrophoresis with a low concentration agar (0.4%). For RNA extractions, freeze-dried green and orange cells were processed following Sanz-Luque and Montaigu (2018) with the only modification being the replacement of the sodium dodecyl sulfate lysis buffer with CTAB.

### Library Preparations and Sequencing

Illumina library preparations and sequencing were done at the Genome Analysis Centre (Earlham Institute, UK) on an Illumina MiSeq with the 500-cycle v2 chemistry kit in 250 bp pair-end mode. PacBio library preparation was performed using SMRTbell Template Prep Kit v1.0, and the sequencing was done on a PacBio Sequel SMRT Cell (2.1 chemistry) at the NERC Biomolecular Analysis Facility, Liverpool. Nanopore library preparation followed the SQK-LSK110 protocol (Oxford Nanopore, Oxford, UK), and sequencing was performed on a MinION with a FLO-MIN106 flow cell, controlled using MinKNOW (19.10.1) at Aarhus University. Raw Nanopore fast5 reads were basecalled with GPU-Guppy (3.2.6+afc8e14) under default settings.

Eukaryotic mRNA was selected from 1 µg of the RNA pool using the NEBNext Poly(A) mRNA Magnetic Isolation Module (New England Biolabs, #E7490) following the manufacturer's instructions. Sequencing libraries were prepared using the NEBNext Ultra II Directional RNA Library Prep Kit for Illumina (New England Biolabs, #E7765) following the manufacturer's instructions, with 5× dilution of the NEBNext Adaptor and 14 library amplifications cycles. The resulting libraries were measured on a Qubit 4 (Thermo Scientific) using the dsDNA High Sensitivity assay and visualized on a Tapestation 4150 (Agilent Technologies) using a D1000 ScreenTape and reagents. Sequencing was performed on an Illumina NextSeq 500 with the 300-cycle v2.5 chemistry kit in 151 bp pair-end mode.

### Genome Assembly, Error Correction, and Contaminant Screening

The qualities of the raw Illumina, Nanopore, and PacBio sequencing reads were inspected using FastQC (Andrews 2010) and RabbitQC (Yin et al. 2021). Adapters and low-quality ends of Illumina reads were trimmed using bbduk.sh script with parameters “k = 23 mink = 11 hdist = 1 tbo trimq = 30 qtrim = rl minlength = 50 maq = 30” (https://sourceforge.net/projects/bbmap/). The basecalled Nanopore reads were processed with Porechop v.0.2.4 (https://github.com/rrwick/porechop) using default parameters to remove sequencing adapters. PacBio and Nanopore long reads were assembled using a hybrid approach with Canu v2.2 (Koren et al. 2017). Two rounds of error correction were performed with Pilon v1.24 (Walker et al. 2014) on the hybrid long-read assembly using the filtered Illumina reads and minimap v2.26 (Li 2018). We assessed the completeness of the assembly based on the Chlorophyta (odb10, 2020-08-05) data set of BUSCO v5.4.6 (Manni et al. 2021), computed summary statistics, and evaluated assembly errors using Inspector v1.0.2 (https://github.com/ChongLab/Inspector).

Assembled contigs were screened for contaminants by assigning taxonomy using National Center for Biotechnology Information (NCBI) Foreign Contaminant Screening tool (FCS-GX; https://github.com/ncbi/fcs-gx). Genome size was estimated using the *k*-mer analysis tool jellyfish v.2.3 (https://github.com/gmarcais/Jellyfish/) using Illumina reads and following an online tutorial (https://bioinformatics.uconn.edu/genome-size-estimation-tutorial/).

### Repeat Masking and Structural and Functional Annotations

A de novo repeat library for strain CCCryo 101-99 was constructed with RepeatModeler v2.0.4 (Smit and Hubley 2008–2015) and RepeatScout v1.0.6 (Price et al. 2005) using the default parameters. First, simple and complex repeats were identified based on the Chlorophyta repeat library of Repbase (version 29.04) using RepeatMasker v4.1.5 (Smit et al. 2013–2015; rmblastn version v2.13.0), and de novo repeats were then identified. Finally, all repeats were combined, and the genome was soft-masked using bedtools v2.26.0 (https://github.com/arq5x/bedtools2/).

Adapter removal and end trimming of Illumina RNA-seq reads were performed using Trimmomatic v.0.39 (Bolger et al. 2014) with parameters “leading: 5 trailing: 5 slidingwindow: 5:10 minlen: 36.” Filtered RNA-seq reads were mapped onto the soft-masked genome using STAR v2.5.2b (Dobin et al. 2013). Coding regions were predicted by incorporating RNA-seq mapping data as extrinsic evidence with BRAKER v3.06 (Stanke et al. 2006, 2008), which is an automated pipeline that utilizes ab initio gene prediction tools GeneMark and AUGUSTUS. The 5′ and 3′ UTR regions were predicted with GUSHR (https://github.com/Gaius-Augustus/GUSHR) based on RNA-seq coverage information, and the predictions were added as gene features to structural annotations. The models and splice patterns were manually checked for a subset of genes with IGV v2.14.1 (Robinson et al. 2011). The longest isoform for each gene was selected for the downstream analyses.

Functional annotations of the BRAKER predicted translations were performed by obtaining the top BLAST hits using BLASTP (*E*-value < 1e^−5^) against SwissProt (UniProt 2023) and DIAMOND BLASTP (https://github.com/bbuchfink/diamond; *E*-value < 1e^−5^, sensitive mode) against UniRef90 (release version: 2023-01) databases. InterPro protein families and domains, Pfam domains, and GO terms were annotated using InterProScan v5.65.97 (Jones et al. 2014). The predictions of the transmembrane domains by TMHMM v2.0c (Krogh et al. 2001) and signal sequences by SignalP v4.1 (Nielsen 2017) were incorporated into InterProScan results. Functional gene enrichment analysis against the *C. reinhardtii* genome was performed using the DAVID web server (https://david.ncifcrf.gov/). The tRNA genes were annotated by tRNAscan-SE v2.0.9 (Chan and Lowe 2019), rRNA genes were annotated using RNAMMER v1.2 (Lagesen et al. 2007), and ncRNA families were annotated using Infernal v1.1.4 (Nawrocki and Eddy 2013) and Rfam database (v14.10). One contig assigned to the chloroplast genome (i.e. based on coverage and GC content) was circularized using a python script (https://github.com/Kzra/Simple-Circularise), annotated using GeSeq (Tillich et al. 2017) and the *C. reinhardtii* chloroplast genome as the reference, and visualized using OGDRAW (Greiner et al. 2019). Default parameters for eukaryotes were used for all tools. All bioinformatic analyses were performed at the GFZ servers in Potsdam.

## Supplementary Material

evae140_Supplementary_Data

## Data Availability

The raw sequences of CCCryo 101-99 are available on NCBI under BioProject accession PRJNA1036577. The Whole Genome Shotgun project has been deposited at DDBJ/ENA/GenBank under the accession JBEFCQ000000000. The version described in this paper is version JBEFCQ010000000. The annotation files are deposited to Zenodo (doi:10.5281/zenodo.10799857). Illumina whole genome sequencing data for CCCryo snow algae strains 002b-99 (*Microglena* sp.), 011a-99 (*Raphidonema sempervirens*), and 005-99 and 047-99 (*Chloromonas remiasii*) are also uploaded under the same BioProject accession. The cultivation and DNA extraction of these strains followed the same protocols as 101-99 as described in the Materials and Methods section. The sequencing of these strains was performed by the Earlham Institute, UK. This additional genome data is currently used by our group for further studies on snow algae.
