# Supplementary material for: Long-Read–Based Hybrid Genome Assembly and Annotation of Snow Algal Strain CCCryo 101-99 (cf. Sphaerocystis sp., Chlamydomonadales)
Source: Genome Biol Evol. 2024 Jun 28;16(7):evae140. doi: 10.1093/gbe/evae140 (PMC11247165; doi:10.1093/gbe/evae140)
Supplement: evae140_Supplementary_Data [file evae140_supplementary_data.docx]

**Supplementary Table 1.** Comparison of the genome assemblies using different error correction strategies and tools

| Assembler | Error correction^α^ | BUSCO (%)  (chlorophyta_odb10,  n=1519) ^β^ | Mapping Rate ^γ^  (%) | Struct. Error^γ^ | Small Scale Error (per 1Mb) ^γ^ | QV score^γ^ | Number of contigs | Total length of contigs longer than 1Mb (Kb) | Total assembly length (Kb) | Contig N_50_ (Kb) | Max. contig length (Kb) | Average GC content (%) |
| --- | --- | --- | --- | --- | --- | --- | --- | --- | --- | --- | --- | --- |
| Canu  (v2.2) | None | C:88.5% [D:0.7%],  F:4.3%, M:7.2% | 81.12 | 1 | 71 | 41.23 | 938 | 50,252 | 119,956 | 806 | 4,582 | 52.9 |
| Canu  (v2.2) | Short reads | C:92.9% [D:0.7%],  F:2.5%, M:4.5% | 81.34 | 1 | 31 | 44.56 | 738 | 55,431 | 122,847 | 855 | 5,321 | 53.1 |
| Canu  (v2.2) | Long + short reads | C:92.7% [D:1.7%],  F:2.4%, M:4.9% | 81.25 | 2 | 107 | 41.23 | 767 | 55,794 | 120,948 | 787 | 3,886 | 53.2 |
| Flye^[[1]](#footnote-1)^  (v2.8.1) | None | C:92.8% [D:0.6%],  F:2.6%, M:4.6% | 79.57 | 5 | 104 | 38.31 | 1152 | 49,176 | 100,480 | 891 | 4,232 | 52.6 |
| Flye^1^  (v2.8.1) | Short reads | C:92.8% [D:0.7%],  F:2.6%, M:4.6% | 80.15 | 2 | 195 | 32.62 | 1105 | 42,754 | 102,676 | 746 | 4,719 | 52.6 |
| Flye^1^  (v2.8.1) | Long + short reads | C:92.9% [D:0.7%],  F:2.6%, M:4.5% | 72.63 | 8 | 305 | 34.85 | 750 | 48,696 | 103,410 | 822 | 4,762 | 52.7 |
| HASLR^[[2]](#footnote-2)^  (v0.8) | None | C:90.2% [D:0.3%],  F:2.8%, M:7.0% | 66.65 | 61 | 925 | 29.27 | 826 | 18,903 | 84,579 | 473 | 1,975 | 52.6 |
| SPAdes^[[3]](#footnote-3)^  (v3.15.5) | None | C:92.4% [D:0.5%],  F:2.6%, M:5.0% | 77.48 | 8 | 159 | 37.64 | 34638 | 10,491 | 10,856 | 167 | 1,888 | 52.6 |

**^α^** One round of correction was performed for each type of read using Pilon.

^β^ C: Complete, D: Complete and Duplicated, F: Fragmented, M: Missing

^γ^ Based on the assessment using Inspector.

**Supplementary Table 2.** Repeat landscape of the CCCryo 101-99 genome

| Repeat category | Number of elements | Length occupied (bp) | Percentage of genome (%) |
| --- | --- | --- | --- |
| Retroelements | 20,988 | 16,216,401 | 13.24 |
| SINEs | 104 | 20,167 | 0.02 |
| Penelope | 1,846 | 538,392 | 0.44 |
| LINEs | 14,604 | 5,358,444 | 4.38 |
| R1/LOA/Jockey | 290 | 87,882 | 0.07 |
| RTE/Bov-B | 8,951 | 1,233,369 | 1.01 |
| L1/CIN4 | 4,793 | 3,275,364 | 2.67 |
| LTR elements | 6,280 | 10,837,790 | 8.85 |
| Ty1/Copia | 271 | 314,982 | 0.26 |
| Gypsy/DIRS1 | 5,835 | 10,295,836 | 8.41 |
| Retroviral | 79 | 45,371 | 0.04 |
| DNA transposons | 1,120 | 612,420 | 0.5 |
| Hobo-activator | 581 | 220,446 | 0.18 |
| Tc1-IS630-Pogo | 78 | 19,441 | 0.02 |
| Rolling circles | 354 | 181,681 | 0.15 |
| Unclassified | 47,537 | 15,888,148 | 12.97 |
| Total interspersed repeats | - | 33,255,361 | 27.15 |
| Small RNA | 335 | 103,156 | 0.08 |
| Simple repeats | 38,671 | 1,873,893 | 1.53 |
| Low complexity | 1,510 | 78,822 | 0.06 |
| Total: | 110,515 | 35,458,147 | 29.98 |

**Supplementary Table 3**. Number and percentage of genes assigned to different gene family, protein domain, and pathway databases.

| Pfam | Swissprot | UniProt | Gene3D domain | Panther family | Signal peptide | Trans-membrane domain | GO term | KEGG pathway | Reactome/  MetaCyc pathway |
| --- | --- | --- | --- | --- | --- | --- | --- | --- | --- |
| 8,536  (56.4%) | 7,689  (51.9%) | 11,137  (75.1%) | 7,685  (51.8%) | 8,805  (59.4%) | 866 (5.8%) | 2,217 (15%) | 7,745 (52%) | 2,946 (%20) | 8,124  (54.8%) |

1. Kolmogorov M, Yuan J, Lin Y, Pevzner PA. 2019. Assembly of long, error-prone reads using repeat graphs. Nat Biotechnol. 37:540–6. [↑](#footnote-ref-1)
2. Haghshenas E, Asghari H, Stoye J, Chauve C, Hach F. 2020. HASLR: Fast Hybrid Assembly of Long Reads. iScience. 23:101389. [↑](#footnote-ref-2)
3. Prjibelski A, Antipov D, Meleshko D, Lapidus A, Korobeynikov A. 2020. Using SPAdes De Novo Assembler. Curr Protoc Bioinformatics. 70:e102. [↑](#footnote-ref-3)
